# A solution based on melatonin, tryptophan, and vitamin B6 (Melamil Tripto©) for sedation in newborns during brain MRI

**DOI:** 10.1186/s13052-019-0714-y

**Published:** 2019-09-23

**Authors:** S. Picone, P. Ariganello, V. Mondì, F. Di Palma, L. Martini, S. Marziali, G. Fariello, P. Paolillo

**Affiliations:** 1 Neonatology and Neonatal Intensive Care Department, Policlinico Casilino General Hospital, Via Casilina 1069, 00169 Rome, Italy; 2 Medical School, La Sapienza University, Piazzale Aldo Moro 5, Rome, Italy; 3 Department of Neuroradiology, Policlinico Casilino General Hospital, Via Casilina, 1069 Rome, Italy

**Keywords:** Melatonin, Neonatal sedation, Brain MRI

## Abstract

**Introduction:**

Melatonin has been studied and used for several years as a sleep-wake cycle modulator in patients with sleep disorders. Experimental evidence has demonstrated the multiple neuroprotective benefits of this indoleamine secreted by the pineal gland. Melatonin is also used in neurological investigations, for its ability to induce sleep in children. In fact, it favors falling asleep during electroencephalogram, Magnetic Resonance Imaging (MRI), and during brainstem auditory evoked potentials. Previous studies are focused on infants and children. No investigation have been performed in neonates, before or during instrumental assessments.

**Material and methods:**

One hundred ten newborns (term and preterm) undergoing brain MRI were enrolled in the study. Thirty minutes before the planned time for the examination, we administered a single dose solution of melatonin- tryptophan-vitamin B6. Twenty minutes after the initial administration of 2 mg, a second dose of 1 mg was administered, if the baby was still awake. If after further 15 min the baby was still not sleeping, an additional dose of 1 mg was administered.

**Results:**

In 106 patients we obtained adequate sedation without adverse events, allowing us to perform an adequate quality MRI, with a median time of 25 min to reach sleeping. Only in three patients MRI could not be performed. In patients having a large weight, higher doses of melatonin were necessary to reach sleeping. Considering the pro kg dose of melatonin, the average dose that induced sleepiness in neonates was 0,64 ± 0.16 mg/Kg.

**Conclusion:**

A solution based on Melatonin- tryptophan-vitamin B6 can be a helpful sedative to administer to neonates undergoing brain MRI, avoiding the use of anesthetics and achieving adequate assessments.

## Introduction

Melatonin (N-acetyl-5-methoxytryptamine) is an endogenous lipophilic indoleamine synthesized by multiple tissues in the body, but the pineal gland is the major contributor to melatonin concentration. In mammals, the melatonin rhythm is generated by an endogenous circadian clock controlled by a paired of nerve cells located just above the optic chiasm in the hypothalamus, called suprachiasmatic nuclei. The biosynthesis of melatonin depends on the availability of tryptophan. Vitamin B6 acts as a co-factor in the conversion of L-5-hydoxy tryptophan to serotonin that is essential for the biosynthesis of melatonin [1, 2].

In neonates circulating plasma melatonin is of maternal origin and its biosynthesis begins between the sixth and eighth week of life [3]. Several lines of evidence suggest a role for melatonin in perinatal disorders, including asphyxia, respiratory distress syndrome, bronchopulmonary dysplasia, surgical interventions, and sepsis [3, 4]. In a recent study, neonates affected by sepsis received orally 20 mg of melatonin, divided in two doses of 10 mg with 1 h interval between the two administrations. It improved the clinical outcome [4]. Post-asphyxia melatonin treatment has also been used at different oral or IV doses to ameliorate neurodevelopmental outcome [5]. Nevertheless, melatonin pharmacokinetics following oral and iv administration in preterm and term newborns is still not completely known. It appears safe, since no side effects were ever reported, even with doses up to 100 mg/Kg administered over 54 h, or with doses of 10 mg/Kg administered once daily for 5 days [6].

Melatonin administration has been reported in children with the aim of sedating patients during Magnetic Resonance Imaging (MRI), Auditory Brain Response (ABR), or electroencephalogram (EEG) recordings, but never in neonates.

In a recent study children undergoing on brain MRI received melatonin at a dose of 10 mg orally, regardless of body weight, 30 min before the examination. All children fell asleep 35 min after melatonin administration. Fifty-five percent of children had a successful MRI. Sleep deprivation improved the success rate. No adverse effects were registered [7].. Another study evaluated sedation with 10 mg oral melatonin to perform MRI in 15 preschool children and satisfactory images were obtained, with no adverse events reported [8]. Wassmer et al. administered melatonin to 27 children at a dosage of 0,25–0,5 mg/kg to perform MRI [9]. Sixteen children were scanned successfully but satisfactory MRI was obtained only in 12. Sury et al. administered either melatonin or placebo 10 min before sedation in addition to standard oral regimen (chloral hydrate (CH) or a combination of temazepam with droperidol) to 98 children aged between 3 months and 10,3 years. The authors concluded that melatonin did not contribute to sedation in children [10].

Sedation with anesthetic drugs is often required in children for EEG registration, requiring the presence of an anesthesiologist to manage possible side effects. In a study performed on 803 children with a mean age of 7,9 years who underwent an EEG sleep recording, 364 patients were administered CH whereas 409 children used a sedation policy consisting in the sequential administration of melatonin, hydroxyzine (if needed) and CH (if needed). With the second sedation policy, the percentage of children requiring CH decreased from 37,1% to 6,7% (*p* < 0.001) [11].

No study has ever evaluated the use of melatonin alone or in combination with tryptophan-vitamin B6, to sedate neonates undergoing instrumental examinations.

We performed the study to assess safety and efficacy of a solution of melatonin- tryptophan-vitamin B6 to sedate both term and preterm neonates undergoing cerebral MRI. We used the solution considering the co-activity of tryptophan and vitamin B6 with the melatonin, the principal inductor sleeping state.

## Material and methods

This is a retrospective study based on our NICU “Brain MRI protocol”. We perform brain MRI in all infants with acute neurological symptoms, particularly with seizures or suspected cerebral malformations, and in Very Low Birth Weight (VLBW) neonates at the age of 40 weeks of postmenstrual age (PMA). Thirty minutes before MRI assessment we administered Melamil Tripto© oral solution, Humana Italia S.p.A, Milan, Italy (containing 1 mg of melatonin, 20 mg of tryptophan, and 1,4 mg of vitamin B6 in 0,5 ml), regardless of body weight.

From November 2017 to February 2019, 110 newborns (73 preterm and 37 term baby) were studied. The sequence of administration, based on our protocol, is the following:

- T0: starting with 1 ml of the solution (corresponding to 2 mg of melatonin);
- T1: 15–20 min form the first administration a further dose of 0,5 ml (1 mg of melatonin, T0 + T1 = 3 mg) is administered only in patients being still awake
- T2: 15 min form the second administration a further dose of 0,5 ml (1 mg melatonin, T0 + T1 + T2 = 4 mg) is administered only in patients being still awake

All the parents signed an informed consent to perform MRI performed as above described. A newborn was excluded for consent withdrawn.

We used a vacuum mattress to contain the infants during MRI examination. It consists of a double waterproof chamber that creates a vacuum with a stiffening of the structure that shapes and immobilizes the patient. The babies wear protective mini muffs to protect them from noise.

Brain MRI examination was performed by a 1.5 T MR System (Prodiva; Philips Medical Systems, Best, The Netherlands) with SENSE head 16-channel coil. Scan protocol included diffusion weighted (DWI) gradient-echo echo-planar (EPI) sequence, axial T1-weighted spin-echo (SE) sequence, axial, coronal and sagittal T2-weighted turbo-spin-echo (TSE) sequence, axial T2*-weighted gradient recalled echo sequence (T2*-GRE), and SWI sequences. In patients with suspected hypoxic-ischemic encephalopathy we added angiographic sequences obtained with the “time of flight” (TOF) technique, and perfusion study was performed using Arterial Spin Labelling technique on the axial plane, without administration of contrast agent, to define ischemic penumbra. Two dedicated neuro-radiologists reported all the examinations and scored the quality of each MRI test on the basis of the value of the images (positive, difficult, negative). The score was positive if it was possible to perform MRI without interruption and with good images; difficult if MRI was performed with frequent interruptions or blurred images; negative if MRI could not be performed.

During examination heart rate (HR) and oxygen saturation (SpO2) was continuously monitored with a pulse oximeter and recorded.

A neonatologist was present at each examination to assess the waking state by a 4 points scale: a) the patient was awake, but tended to fall asleep if left alone; b) the patient could wake up if stimulated, but tended to be asleep; c) the patient was awake; d) other.

Once back in the ward, the patient was continuously monitored with an oximeter and hearth rate monitor.

Statistical analysis was performed using 1- way ANOVA (Kruskal–Wallis tests) and X^2^ (R-Pearson test) as appropriate. *p* < 0.05 was considered significant. Data are presented as media and deviation standard (DS) or medians and interquartile ranges (IQR), if the variables is normally distributed or not.

## Results

In our study we have retrospectively analyzed data of 110 infants (73 preterm and 37 term baby) who needed to undergo MRI, in a period of 16 months. A newborn was excluded for consent withdrawn.

Table 1 shows collected data of 109 neonates who performed the assessment (1 was a drop-out), as GA, BW, PMA at MRI, weight at MRI. Considering all the neonates, the median time to achieve sleep and to start the MRI was 25 min (IOR 15–35). The median duration of the exam was also 25 min (IQR 20–35). The heart rate remained between 130 and 145 (median 140 bpm) and SpO2 between 97 and 99% (median 98%). SpO2 and HR were also registered returning to NICU, and no significant alteration of these parameters were reported.

**Table 1:** Collected data

	median	IQR
GA	31^+ 2^	30–37^+ 5^
BW	1440	1252–2020
PMA at MRI	46^+ 1^	42^+ 6^–48^+ 4^
Weight at MRI	4400	3640–5040
Time to start MRI	25	15–35
Duration MRI	25	20–35
HR	140	130–145
SatO2	98	97–99
Dose (mg) pro Kg (median ± ΔS)	0,64 ± 0,16	

Gestational age (GA, weeks), birth weight (BW, g), postmenstrual gestational age (PMA, weeks) on time of MRI and weight at time at MRI, time needed for the subject to fall asleep before test (time interval between administration and the onset of MRI) (minutes), exam duration (minutes), heart rate (bpm) and oxygen saturation (StaO2) (%) during the MRI examination

We highlighted that the average melatonin dose pro Kg administered was 0,64 ± 0,16 mg/Kg. No side effects were reported.

One ml of the solution (corresponding to 2 mg melatonin) was administered to 32 patients; 1,5 ml (3 mg) to 72 patients and 2 ml (4 mg) to 5 infants. The patients were divided in the three groups, related to the dose of melatonin administered (Table 2). There were no significant differences between the groups comparing GA, BW, PMA at MRI examination, HR, and SatO2. Even if there is no significant difference comparing the median time to reach sleeping between the three groups, the interval between administration and falling asleep was longer in neonates of higher weight (until 35 min in the 3 mg group and until 50 min in the 4 mg group), with no significant difference between the groups (*p* = 0,52). There is a significant difference (*p* < 0.001) between the dose per Kg (mg/Kg) of melatonin between the three groups: A higher dose was needed in patient with higher weight (an average of 0,52 mg in infants with an average weight of 3800 g vs an average dose of 0,85 mg in neonates with an average weight of 4600 g). (Table 2).

**Table 2:** Demographic and during MRI characteristic of the patients enrolled into the study, comparing the 3 groups related do the admisnistred dose of melatonine

Dose (number of neonates)	2 mg (32)	3 mg (72)	4 mg (5)	*p*-value
GA (wk) median (IQR)	31,1 (29,7-30)	31,3 (29,7-36,2)	31,1 (27,9-39,5)	1
BW (g) median (IQR)	1300 (1025-2665)	1530 (1310-2040)	1300 (1030-1300)	0,058
PMA (wk) at MRI median (IQR)	46 (42,3-48,7)	47 (42,9-48,9)	44 (43,7-47,4)	0,69
Weight at MRI (g) median (IQR)	3800 (3190-4600)	4500 (3800-5200)	4600 (3740-5975)	*0,017*
Time (min) to reach sleeping median (IQR)	25 (15-35)	25 (17-35)	32 (22-50)	0,52
Duration MRI (min) median (IQR)	20 (20-30)	25 (17-35)	31 (26-34)	0,42
HR (bpm) median (IQR)	140 (130-148)	140 (130-145)	142 (132-143)	0,66
SatO2 (%) median (IQR)	98 (97-98)	98 (97-99)	99 (96-99)	0,24
Dose pro Kg (mg/kg) median (DS)	0,52 (± 0,11)	0,64 (± 0,15)	0,85 (± 0,16)	*< 0,001*

*GA* Gestational Age, *BW* Birth Weight, *PMA* Post Menstrual Age, *HR* Heart Rate, *IQR* Interquartile Range, *DS* Deviation Standard

Table 3 reports outcome of MRI based on the quality of the MRI images and the mode of the waking state of the neonates, in relation to the 3 doses of administered melatonin. In 3 patients (all sedated with 3 mg of melatonin) was no possible to complete MRI examination because they woke up in course of the radiological session.

**Table 3:** Effect of melatonin administration during MRI performing and patient evaulation after MRI

	2 mg (32)	3 mg (72)	4 mg [5]	*p*-value
Quality of MRI				
Positive (patient, %)	26 (81,2%)	67 (93%)	5 (100%)	
Diffucult (patient, %)	6 (17,8%)	2 (2,7%)	0 (0%)	
Negative (patient, %)	0 (0%)	3 (4,3%)	0 (0%)	
				*0,04*
Waking state after MRI				
Awake, tends to fall asleep	10 (31,2%)	16 (22,2%)	0 (0%)	
Awake if stimulated	3 (9,3%)	7 (9,8%)	0 (0%)	
Awake	19 (59,5%)	49 (68%)	5 (100%)	
				0,6

Moreover, in all the patients who took 4 mg of the solution, difficulty in performing the MRI was reported. In patients sedated with 2 or 3 mg, the quality of MRI was positive in 81,2 and 93%, respectively (*p* = 0.04).

## Discussion

Premature infants, especially the extremely low birth weight (ELBW), are at high risk for impaired neurological outcome. Cerebral ultrasound, though performed regularly, has a poor sensitivity to assess a prognosis of neurodevelopmental impairment, while MRI at term equivalent age may improve prognostication and identification of those at highest risk for impairment [12, 13]. In addition, in our NICU, MRI is indicated in neonates with acute pathology such as in the presence of seizures, in hypoxic-ischemic encephalopathy, or brain malformations. Previously, we have observed that the use of the vacuum mattress alone was not always sufficient to obtain the necessary complete immobilization of the neonate, causing an extension of the examination (about 1 h). In literature many different strategies have been described to provide patient immobility during an MRI in the newborn population in order to decrease image artifacts due to motion, including general anesthesia. However, there is a growing body of evidence, especially in animal studies, that links neurotoxicity to anesthetic exposure in the developing brain [14, 15]. The UK National Institute for Clinical Excellence (NICE) recommends the use of oral chloral hydrate in children under 15 kg [16], yet some studies have shown that chloral hydrate can cause an increase in episodes of bradycardia, apnea, and reduced oxygen saturation [17]. These complications have been postulated to be more frequent in infants with lower birth weight [18].

Moreover, a pharmacological sedation requires the presence of the anesthesiologist, with considerable management problems. This is the reason why, from November 2017, we have included in our “MRI protocol” the use of a solution with melatonin-tryptophan-vitamin B6 to improve sedation during MRI execution*.* Melatonin has been administered in children for some years as a sleep inducing agent in neurophysiological and neuroimaging procedures like EEG, ABR, and MRI. All the studies were conducted in children over 1 year of age.

To our knowledge only few investigations have been done to evaluate children performing instrumental assessment with the same solution we tested in our study. It has been used in 294 children (average age 29.8 months) who underwent ABR. Three different dosages were tested: two different administration timings (pre-treatment and single shot treatment) and three dosages (0.5 ml, corresponding to 1 mg of melatonin, in pre-treatment, 1.5 ml, corresponding to 3 mg of melatonin, in pre-treatment, and 3 ml, corresponding to 6 mg of melatonin in single shot). The authors found a more rapid induction of sleep using pre-medication with 1.5 ml solution (3 mg of melatonin), with a reduction in the duration of the exam [19, 20].

Our study shows that an oral solution of melatonin-tryptophan-vitamin B6 is a useful sedative to properly perform brain MRI in infants. The administration of 2–4 mg of melatonin with tryptophan and vitamin B6, on average 25 min before the examination, allowed in all the patients to perform the examination correctly and quickly. Even if there are no significant differences in the timing of starting and ending the brain MRI, the higher dose necessary in the patient with higher weight, could suggest the need to establish a minimum and maximum dose per Kg in neonates. In our experience the average dose pro kg of melatonin to get sleep was of 0,64 ± 0,16 mg/kg in a single dose about 25 min before the exam, with a successful rate (positive quality of MRI) of 89,9% of total patients (98/109).

Moreover, no side effects have been reported up to 4 mg. The use of this solution allowed us to avoid the administration of sedative drugs, therefore avoiding the need of the presence of the anesthesiologist during MRI.

Further investigations are required to confirm these promising results.

## Conclusion

Our data support that an oral solution based on Melatonin- tryptophan and vitamin B6 (Melamil Tripto©) is a useful sedative that allows to adequately perform brain MRI in term and preterm newborns, avoiding sedation with drugs that may have serious side effects and require anesthesiologists during the examination. An oral dose of 2–4 mg of melatonin- tryptophan-vitamin B6 solution in newborns who underwent MRI allowed to complete successfully the examination in most of the cases, with higher doses needed in the high-weight infants. However, more studies are needed to standardize dosage in relation to the body weight in neonates.

## Data Availability

All clinical data and material are available in our Unit.

## Abbreviations

ABR: Auditory Brain Response
CH: Chloral hydrate
DS: Deviation Standard
EEG: Electroencephalogram
ELBW: Extremely low birth
HR: Heart rate
IQR: Interquartil Range
MRI: Magnetic Resonance Imaging
PMA: Postmenstrual age
VLBW: Very Low Birth Weight
